# Documented Valvular Regurgitation and Explicit Heart Failure in an Internal Medicine Registry: A Cross-Sectional Study

**DOI:** 10.3390/medicina62101835

**Published:** 2026-09-23

**Authors:** Maria Rada, Maria-Laura Craciun, Ana-Maria Pah, Diana Utu, Dana Emilia Velimirovici, Diana-Maria Mateescu, Ioana Cotet, Ariana Bianca Velciov, Cristiana Adina Avram

**Affiliations:** 1 Department VI, Cardiology, Faculty of Medicine, “Victor Babeș” University of Medicine and Pharmacy Timișoara, Eftimie Murgu Square 2, 300041 Timișoara, Romania; rada.maria@umft.ro (M.R.); laura.craciun@umft.ro (M.-L.C.); dana.velimirovici@umft.ro (D.E.V.); 2 Center for Preventive Medicine, Faculty of Medicine, “Victor Babeș” University of Medicine and Pharmacy Timișoara, Eftimie Murgu Square 2, 300041 Timișoara, Romania; 3 Department II, Physiology and Pathophysiology, Faculty of Pharmacy, “Victor Babeș” University of Medicine and Pharmacy Timișoara, Eftimie Murgu Square 2, 300041 Timișoara, Romania; 4 Department of Infectious Diseases, Municipal Emergency Clinical Hospital Timișoara, 2A Hector Street, 300041 Timișoara, Romania; diana.mateescu@umft.ro; 5 Doctoral School, “Victor Babeș” University of Medicine and Pharmacy Timișoara, Eftimie Murgu Square 2, 300041 Timișoara, Romania; ioana.cotet@umft.ro; 6 Food Science Department, University of Life Sciences “King Michael I” of Timișoara, Calea Aradului 119, 300645 Timișoara, Romania; arianavelciov@usvt.ro; 7 Department of Internal Medicine I, Faculty of Medicine, “Victor Babeș” University of Medicine and Pharmacy Timișoara, Eftimie Murgu Square 2, 300041 Timișoara, Romania; avram.adina@umft.ro

**Keywords:** valvular regurgitation, valvular heart disease, heart failure, NYHA class, clinical registry, reverse causation, ascertainment bias, occupational cohort, cross-sectional study

## Abstract

*Background and Objectives*: Routine records may conflate valvular heart disease (VHD), functional classification, and shared diagnostic assessment. We quantified the cross-sectional association between a registry VHD label and explicitly documented heart failure (HF), while recognizing that every observed valve lesion was regurgitant. *Materials and Methods*: The analytic sample comprised all 224 eligible adults admitted to the Internal Medicine II Department of a railway-affiliated hospital between 1 August 2025 and 31 July 2026: 38 with documented VHD and 186 without documented VHD. The primary outcome was an explicit HF diagnosis; a combined HF/NYHA-positive field was examined as an over-inclusive sensitivity outcome. Logistic regression was adjusted for age, sex, body mass index, smoking history, and total cholesterol. *Results*: Explicit HF was documented in 24/38 patients with VHD (63.2%) and 72/186 without VHD (38.7%; adjusted OR 2.87, 95% CI 1.38–5.99). The combined outcome was present in 28/38 (73.7%) and 101/186 (54.3%), respectively (adjusted OR 2.38, 95% CI 1.09–5.21). All 38 exposed patients had documented mitral, aortic, and/or tricuspid regurgitation; no stenosis, prolapse, or prior valve intervention was recorded. *Conclusions*: Documented valvular regurgitation was associated with explicit HF documentation. Because regurgitation may be secondary to ventricular or atrial disease, reverse causation, shared ascertainment, non-adjudicated diagnoses, and residual confounding preclude causal, temporal, prognostic, or lesion-specific inference.

## 1. Introduction

Valvular heart disease (VHD) is a heterogeneous source of cardiovascular morbidity worldwide [1,2]. Contemporary guidelines emphasize that lesion severity, symptoms, ventricular response, pulmonary pressures, rhythm, and the consequences of pressure or volume overload determine clinical risk and the timing of intervention [1]. Heart failure (HF) is a major manifestation of advanced valve disease, but the relationship is often bidirectional: structural valve lesions can drive adverse remodeling and congestion, whereas ventricular or atrial disease can produce secondary mitral or tricuspid regurgitation [3,4,5,6,7].

Routine clinical registries seldom capture these mechanisms with the completeness of dedicated imaging cohorts. A documented VHD label may therefore represent a mixture of lesion types, severities, prior interventions, referral patterns, and diagnostic intensity. Similarly, a recorded HF diagnosis may not distinguish incident from prevalent disease or HFrEF, HFmrEF, and HFpEF. Analyses of such data should consequently estimate co-occurrence without implying temporal direction or causality.

Two mechanisms may contribute to an association between these diagnostic labels in registry data, and conventional covariate adjustment cannot distinguish them. Valve disease may contribute to congestion and HF, while shared clinical assessment may increase the likelihood that both conditions are documented during the same episode of care. Echocardiography is central to contemporary VHD assessment and is commonly obtained when HF is suspected or established. A cross-sectional analysis of routine records should therefore be interpreted as documented co-occurrence potentially influenced by diagnostic intensity, rather than as a direct estimate of a causal effect.

The primary objective was to quantify the cross-sectional association between the registry VHD exposure and explicitly documented HF. Secondary objectives were to determine whether the association persisted when the over-inclusive combined HF/NYHA-positive field was used, to describe the observed valve-territory pattern, and to report outcome frequencies separately by exposure status. Because every eligible comparator was retained, the exposed fraction among eligible adults can also be reported as a registry-specific documentation frequency. These analyses distinguish a potentially useful co-documentation signal from associations produced by functional classification, selection, reverse causation, or shared diagnostic assessment.

## 2. Materials and Methods

### 2.1. Study Design and Reporting Framework

This retrospective cross-sectional analysis used a clinical registry from the Internal Medicine II Department of the Clinical CF Hospital Timișoara (Spitalul Clinic Căi Ferate). The hospital serves primarily employees of Căile Ferate Române (Bucharest, Romania), the Romanian state railway carrier, together with eligible family members and other insured patients. All patients included in the present analysis were admitted to the Internal Medicine II Department. This occupational catchment, rather than a general community cardiology service, accounts for the predominantly male, mid-adult to early-retirement age profile of the sample. The registry contained demographic, anthropometric, smoking, laboratory, and cardiovascular diagnostic information recorded during hospital admissions between 1 August 2025 and 31 July 2026. Reporting was prepared with reference to the STROBE recommendations for cross-sectional studies [8]. The completed checklist is provided in Supplementary Table S1.

### 2.2. Source Population and Analytic Cohort

A total of 312 unique adult patients admitted to the Internal Medicine II Department between 1 August 2025 and 31 July 2026 were screened (Figure 1). Identifiers were used solely for patient-level deduplication and consent verification and were removed before analysis. Eligibility was assessed without reference to HF/NYHA status and required documented research consent, interpretable VHD and HF/NYHA fields, a plausible BMI, an interpretable smoking code, and a total cholesterol value within 80–400 mg/dL. Criteria were applied sequentially in this order, and each excluded patient was counted once, under the first criterion not met. Eighty-eight patients were excluded: missing documented research consent (*n* = 14), uninterpretable or missing VHD or HF/NYHA fields (*n* = 18), implausible or missing BMI (*n* = 11), uninterpretable smoking code (*n* = 9), and missing or out-of-range total cholesterol (*n* = 36). The complete eligible cohort therefore comprised 224 unique adults: all 38 eligible patients with documented VHD and all 186 eligible patients without documented VHD. No sampling and no outcome-dependent selection were performed.

An earlier archived extract had retained all 38 VHD patients and a random subset of 72 comparators from the same eligible non-VHD pool. After peer review, the registry was re-queried to recover the complete eligible cohort and the full participant flow; the present analysis supersedes that extract. The total-cholesterol requirement originated in the historical complete-core-record definition and is not biologically necessary for defining VHD or HF. It was retained for the primary analysis so that crude and adjusted estimates used the same participants without imputation, and its potential for selection bias was examined in a sensitivity analysis that re-included the 36 patients excluded at that final step (Section 2.6 and Section 3.5; Supplementary Table S2, Panel C). Because the cohort is the complete eligible set rather than an exposure-enriched subsample, group-specific HF frequencies, the odds ratio, and the eligible-cohort VHD fraction (38/224) are interpretable as documentation frequencies within this occupational inpatient registry. They do not estimate community or outpatient-cardiology prevalence.

### 2.3. Definition and Observed Composition of the Valvular Exposure

The registry VHD variable was ascertained from cardiovascular diagnosis fields and normalized free-text terms prespecified to capture mitral, aortic, or tricuspid regurgitation/insufficiency, stenosis, prolapse, degenerative valve disease, or a documented history of valve repair or prosthetic-valve treatment accompanied by a valvular diagnosis. In the analyzed cohort, however, every positive exposure record contained regurgitation/insufficiency terminology: no stenosis, mitral valve prolapse, degenerative lesion without regurgitation, valve repair, or prosthetic valve was documented. The observed exposure is therefore more precisely interpreted as documented valvular regurgitation recorded under the registry VHD label, rather than VHD across its usual lesion spectrum. More than one valve territory could be assigned to the same patient; multivalvular involvement was defined as documentation in at least two territories. Uniform quantitative echocardiographic measurements were unavailable. Consequently, severity, mechanism, etiology, primary versus secondary regurgitation, LVEF, and HF phenotype could not be re-adjudicated. Documentation may reflect structural disease, ventricular or atrial remodeling, prior investigation, referral pattern, and diagnostic intensity.

### 2.4. Heart Failure Outcome

The primary outcome was explicitly documented HF, defined as a formally recorded HF diagnosis in the source record. A positive NYHA entry without an explicit HF diagnosis was not considered HF for the primary analysis because NYHA class is a functional classification rather than a diagnosis [3,4,9]. As a sensitivity analysis, we used the original over-inclusive HF/NYHA-positive registry field, which combined explicit HF with any positive entry in the associated NYHA field. NYHA-only patients remained in their exposure groups and were classified as primary-outcome negative but combined-outcome positive. Among patients with VHD, 24 had explicit HF, 4 had NYHA-only positivity, and 10 had neither. Among patients without VHD, 72 had explicit HF, 29 had NYHA-only positivity, and 85 had neither. LVEF was not systematically available, precluding reproducible classification as HFrEF, HFmrEF, or HFpEF.

### 2.5. Covariates

Age and sex were taken from the structured registry fields. BMI was used when a plausible value was directly recorded; otherwise, it was calculated as body weight in kilograms divided by height in meters squared. Smoking was analyzed as a binary documented smoking history (yes/no): a zero registry code was treated as no documented smoking, whereas any nonzero code was treated as a positive smoking history. This conservative mapping avoided inferring current-versus-former smoking because the registry coding did not consistently distinguish these categories.

Total cholesterol was expressed in mg/dL; a value within the plausibility range of 80–400 mg/dL was an eligibility requirement for the primary analysis (Section 2.2). Total cholesterol was included as a covariate in the adjusted models but is not conceptually required to define the VHD–HF question. Other lipid fractions (triglycerides and high- and low-density lipoprotein cholesterol) were inconsistently recorded, were not part of the eligibility rules or the analytic models, and were not analyzed.

### 2.6. Statistical Analysis

Continuous variables are summarized as median [interquartile range (IQR)] and categorical variables as *n* (%). Between-group comparisons used the Mann–Whitney U test for continuous variables and Fisher’s exact test for categorical variables. 

Binary logistic regression evaluated the association between the registry VHD exposure and each HF outcome. The prespecified primary model used explicitly documented HF. Unadjusted models included VHD alone; adjusted models included VHD, age (per 10 years), sex, BMI (per 5 kg/m^2^), documented smoking history, and total cholesterol (per 10 mg/dL), the structured covariates available for every participant. The combined HF/NYHA-positive field was analyzed with the same adjustment set as an outcome-definition sensitivity analysis. A second sensitivity analysis re-included the 36 patients excluded only for missing or out-of-range total cholesterol (extended cohort, *n* = 260). Because that analysis specifically assessed the effect of the cholesterol exclusion, extended-cohort associations were estimated from 2 × 2 tables using unadjusted binary logistic regression. Odds ratios and Wald 95% confidence intervals were calculated from observed cell counts; two-sided Wald *p* values are reported (Supplementary Table S2).

Major cardiovascular confounders—including coronary artery disease, atrial fibrillation, hypertension, diabetes, chronic kidney disease, prior valve intervention, and treatment exposure—were unavailable. No a priori power calculation was performed because the study used the complete eligible cohort. All tests were two-sided, with *p* < 0.05 considered statistically significant. Analyses were performed using SPSS version 29.0 (IBM Corp., Armonk, NY, USA). The primary explicit HF model and all sensitivity analyses were complete-case analyses; no imputation was performed.

### 2.7. Ethics

The study was conducted in accordance with the Declaration of Helsinki and was approved by the Ethics Council of the Clinical CF Hospital Timișoara (approval no. 5966, issued 30 July 2026). The approval covers review of records from the Internal Medicine II Department for the period from November 2024 through 31 July 2026. The narrower analytic admission window used in this study was 1 August 2025 through 31 July 2026 and should not be confused with the broader approved data window. All data extraction, deduplication, eligibility assessment, and analysis were conducted within the scope of this approval. Written informed consent for research use of anonymized clinical data was documented at admission and verified before de-identification.

## 3. Results

### 3.1. Study Population and Valvular Phenotype

Of 312 screened patients, 88 were excluded and 224 unique eligible adults were analyzed: 38 with a registry VHD label and 186 without (Figure 1). The exposed group therefore accounted for 38/224 eligible adults (17.0%) in this occupational inpatient registry. Median age in the exposed group was 63 [52.3–69.0] years, and 30/38 (78.9%) were male. Mitral regurgitation/insufficiency was documented in 33/38 exposed patients (86.8%), aortic regurgitation in 9/38 (23.7%), and tricuspid regurgitation in 3/38 (7.9%); seven patients (18.4%) had at least two valve territories recorded. No stenosis, mitral valve prolapse, degenerative lesion without regurgitation, prior valve repair, or prosthetic valve was documented. Thus, despite the broader prespecified extraction definition, the observed exposure consisted exclusively of documented valvular regurgitation.

### 3.2. Primary Explicit HF Outcome and Baseline Characteristics

Explicit HF was documented in 24/38 patients with the VHD exposure (63.2%) and 72/186 without it (38.7%; Fisher’s exact *p* = 0.007). The unadjusted OR was 2.71 (95% CI 1.32–5.59; *p* = 0.007). The combined HF/NYHA-positive sensitivity outcome was present in 28/38 (73.7%) and 101/186 (54.3%), respectively (Fisher’s exact *p* = 0.031; unadjusted OR 2.36, 95% CI 1.08–5.13). Four and 29 patients, respectively, qualified for the combined field through NYHA documentation alone. Across the cohort, explicit HF was recorded in 96/224 (42.9%), and the combined field was positive in 129/224 (57.6%). BMI did not differ between exposure groups (26.0 [23.3–27.0] vs. 26.1 [23.5–28.2] kg/m^2^; *p* = 0.512), unlike the imbalance observed in the earlier comparator subsample, further supporting use of the complete eligible cohort. The complete baseline characteristics according to registry-defined VHD exposure are presented in Table 1.

### 3.3. Primary Association with Explicitly Documented Heart Failure

For the primary explicit HF outcome, the adjusted OR for the registry VHD exposure was 2.87 (95% CI 1.38–5.99; *p* = 0.005), compared with an unadjusted OR of 2.71 (95% CI 1.32–5.59; *p* = 0.007). Age, sex, BMI, smoking history, and total cholesterol were not associated with explicit HF at conventional thresholds (Table 2). The adjusted estimate is a conditional association between recorded labels and must not be interpreted as an independent or causal effect.

### 3.4. Outcome-Definition Sensitivity Analysis Using the Combined HF/NYHA Field

The combined HF/NYHA-positive field included explicit HF plus 33 patients with NYHA-only positivity. It was present in 28/38 exposed patients (73.7%) and 101/186 unexposed patients (54.3%; Fisher’s exact *p* = 0.031). The unadjusted OR was 2.36 (95% CI 1.08–5.13; *p* = 0.031), and the adjusted OR was 2.38 (95% CI 1.09–5.21; *p* = 0.030). The similarity to the stronger primary explicit HF estimate indicates that the association was robust to inclusion of NYHA-only positivity and was not generated by the 33 NYHA-only records (Supplementary Table S2).

### 3.5. Sensitivity Analysis Including Patients Excluded for Total Cholesterol

The extended cohort comprised 260 patients: 44 with documented VHD and 216 without documented VHD. The combined HF/NYHA-positive field was present in 32/44 (72.7%) and 115/216 (53.2%) patients, respectively, and explicit HF in 27/44 (61.4%) and 82/216 (38.0%). For the combined outcome, the unadjusted OR for documented VHD was 2.34 (95% CI 1.15–4.79; *p* = 0.020). For explicit HF, the unadjusted OR was 2.60 (95% CI 1.33–5.05; *p* = 0.005). These estimates were calculated directly from the reported cell counts using the prespecified Wald method and were similar to the corresponding unadjusted estimates in the primary cohort of 224 patients (OR 2.36, 95% CI 1.08–5.13 for the combined outcome; OR 2.71, 95% CI 1.32–5.59 for explicit HF). Re-including patients excluded for total cholesterol therefore did not materially change the direction or magnitude of the unadjusted association.

## 4. Discussion

The primary analysis showed an association between the registry VHD exposure and explicitly documented HF (63.2% vs. 38.7%; crude OR 2.71, 95% CI 1.32–5.59; adjusted OR 2.87, 95% CI 1.38–5.99). The association persisted when the over-inclusive combined HF/NYHA-positive field was used (adjusted OR 2.38, 95% CI 1.09–5.21). Importantly, every observed valve lesion was regurgitant; no stenosis, prolapse, or prior valve intervention was documented. The finding is therefore best interpreted as a co-documentation signal between valvular regurgitation and HF, with reverse causation and shared ascertainment as prominent competing explanations.

### 4.1. Interpretation of the Primary Finding

The direction of association is clinically plausible in both directions. Primary valvular regurgitation can produce volume overload, remodeling, and HF, whereas ventricular or atrial disease can generate secondary mitral or tricuspid regurgitation [1,3,4,5,6,7]. The absence of stenosis and the exclusively regurgitant exposure shift the balance of plausible explanations toward HF-related remodeling and reverse causation compared with a VHD exposure containing primary stenotic lesions. Because exposure and outcome were recorded cross-sectionally and lesion mechanism was unavailable, the data cannot determine temporal direction.

Although the extraction dictionary was designed to capture the broader VHD spectrum, the analyzed exposure consisted only of mitral, aortic, and/or tricuspid regurgitation. Two non-exclusive explanations are plausible. First, this occupational inpatient cohort was younger than many referral-based VHD populations and may contain fewer calcific stenotic and previously intervened lesions. Second, normalization of routine free text may preferentially capture commonly recorded regurgitation terminology, whereas stenosis, prolapse, or intervention history may be under-recorded outside a dedicated echocardiography registry. Without uniform imaging reports, neither explanation can be quantified. The findings must therefore not be generalized to stenotic, prolapsing, prosthetic, or post-intervention valve disease.

Mitral involvement predominated (86.8%), and 18.4% of exposed patients had at least two valve territories documented. Published multivalvular estimates vary with setting and definition [10,11,12,13]. However, direct comparison is limited because the current exposure was exclusively regurgitant, non-adjudicated, and recorded in routine internal medicine data. HF documentation was also frequent among exposed patients (63.2% for explicit HF; 73.7% for the combined field), consistent with inpatient shared ascertainment and with the possibility that ventricular or atrial disease produced secondary regurgitation [5,6,14,15,16,17]. Mechanism and severity were unavailable, so the observed territory pattern should not be compared directly with imaging-adjudicated cohorts [18,19,20].

Etiology is clinically important because primary and secondary regurgitation have different relationships with HF. Primary degenerative, rheumatic, congenital, or infective valve disease may lead to volume overload and HF, whereas ischemic, ventricular, or atrial remodeling may cause functional regurgitation after or alongside HF. An etiologic subgroup analysis was not performed because standardized imaging and etiologic adjudication were unavailable and the 38 exposed patients formed small, overlapping territory groups. Assigning mechanism from non-specific routine text would be unstable and potentially misleading. Future registries should prospectively distinguish primary from secondary regurgitation and separately capture stenosis, prolapse, prosthetic valves, and prior intervention [1,3,4,5,6,9,18,19,20,21,22,23,24].

Age requires careful interpretation. Clinically important VHD rises steeply with age, but the exposed group had a median age of 63 years in this predominantly male occupational cohort. This younger case-mix may partly explain the absence of calcific stenosis and prior valve intervention, although preferential recording of regurgitation in free text remains an alternative explanation. Age showed little association with either recorded outcome, which should not be interpreted as evidence that age is clinically unimportant. Restricted age distribution, modest sample size, shared ascertainment, and unavailable age-related cardiovascular confounders limit inference [2,10,11,23].

### 4.2. Shared Ascertainment as a Competing Explanation

Shared ascertainment is a central competing explanation. Evaluation for suspected HF may increase the probability that valve disease is investigated and documented, while a known valve lesion may prompt more intensive assessment for HF. Because the registry did not record whether, when, or why echocardiography was performed, diagnostic intensity cannot be separated from structural co-occurrence in the observed OR.

Adjustment for age, sex, BMI, smoking, and total cholesterol cannot correct unmeasured testing, referral, or documentation processes. In addition, clinically relevant valve disease may remain undocumented in patients who undergo less intensive imaging [11]. The direction and magnitude of these ascertainment and misclassification effects are unknown; accordingly, the adjusted estimates are reported only as conditional associations between routinely recorded labels.

### 4.3. Outcome Definition and Residual Confounding

The main limitations are reverse causation, shared ascertainment, non-adjudicated diagnoses, exposure-spectrum restriction, and residual confounding. Explicitly documented HF was selected as the primary outcome because it is more diagnostically defensible than NYHA positivity. The combined-field sensitivity analysis produced a similar but weaker association, supporting robustness to outcome definition. Nevertheless, an explicit chart label is not independent HF adjudication. The absence of standardized echocardiography prevented confirmation of regurgitation severity and mechanism and may have missed stenotic or previously treated disease. The results apply to documented regurgitation in this registry, not to VHD generally.

All 186 eligible non-VHD comparators were included in the revised analysis, eliminating the sampling-efficiency concern associated with the earlier random subset. The largest exclusion step was missing or out-of-range total cholesterol (36/312 screened patients); re-including these patients yielded similar unadjusted estimates (Section 3.5), reducing—but not eliminating—concern about selection from that criterion. The occupational, predominantly male inpatient case-mix limits generalizability to settings with comparable referral and documentation practices.

### 4.4. Clinical and Research Implications

The primary association between documented valvular regurgitation and explicit HF supports more systematic verification and structured phenotyping in routine registries. A practical minimum dataset would record explicit HF separately from NYHA class and capture valve territory, lesion type, mechanism and severity, LVEF-based HF phenotype, ventricular dimensions and function, pulmonary pressures, rhythm, congestion, major cardiovascular and renal comorbidities, prior valve intervention, and treatment. In clinical workflows, co-documentation can prompt confirmation that contemporary echocardiography and phenotype documentation are complete. This is a quality-of-documentation implication, not evidence that any screening or treatment strategy improves outcomes.

Prospective registries should predefine imaging intervals, apply standardized lesion and HF criteria, and distinguish primary from secondary regurgitation. Independent HF adjudication and longitudinal follow-up would then permit lesion-specific, prognostic, and causal questions that the present cross-sectional dataset cannot answer. The current estimates are suitable for coding audit and hypothesis generation, not individual risk prediction or treatment selection.

## 5. Conclusions

In this complete eligible inpatient registry cohort, documented valvular regurgitation was associated with explicitly documented HF (adjusted OR 2.87, 95% CI 1.38–5.99). The association remained when the over-inclusive combined HF/NYHA-positive field was used as a sensitivity outcome (adjusted OR 2.38, 95% CI 1.09–5.21). Because the exposure contained no stenosis, prolapse, prosthetic valve, or prior intervention, reverse causation through ventricular or atrial disease causing secondary regurgitation is a particularly important competing explanation. The findings represent co-documentation, not a causal, temporal, prognostic, or lesion-specific effect, and do not generalize to the full VHD spectrum.

## Figures and Tables

**Figure 1:**
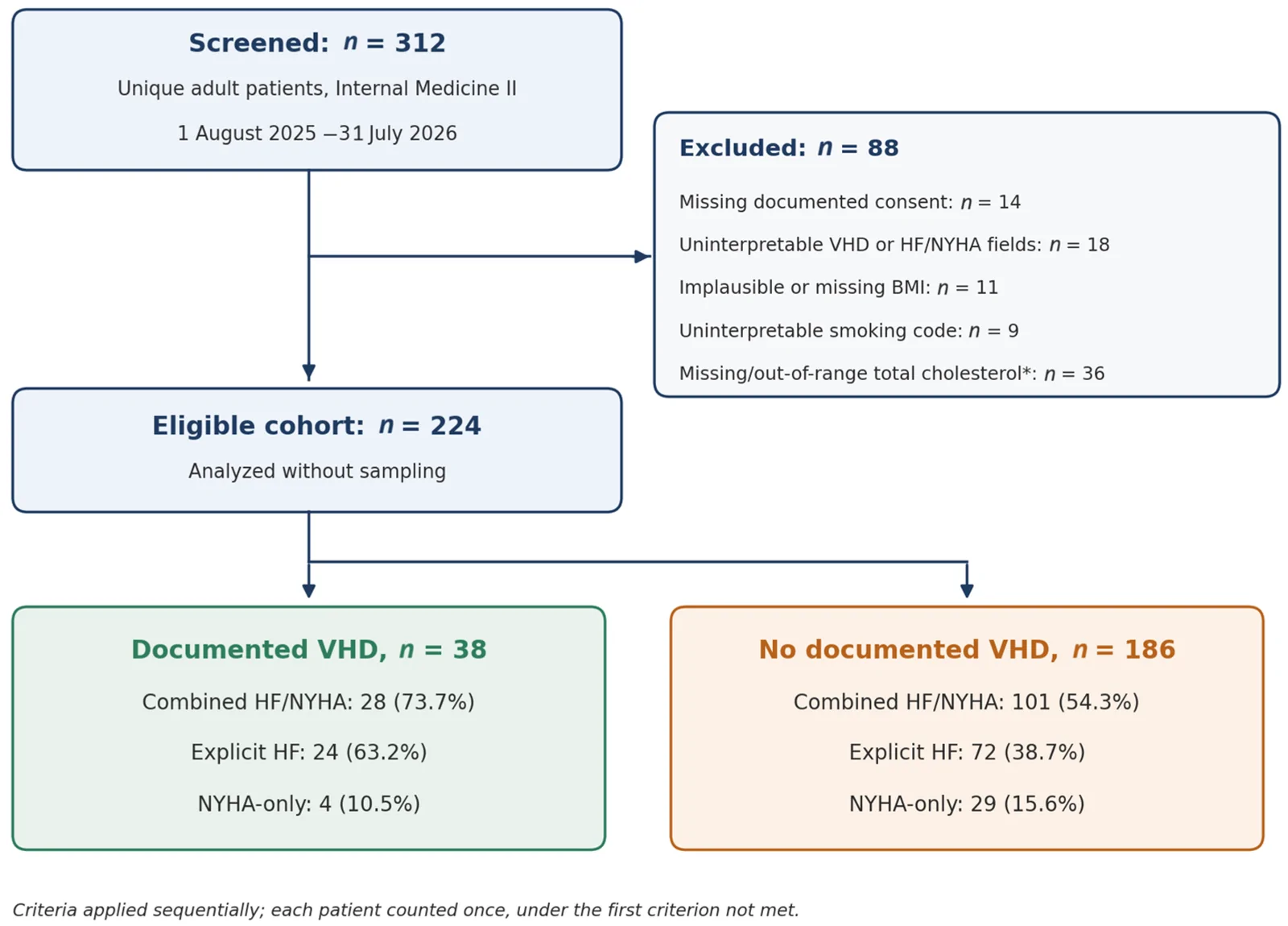
Participant flow from screening to the complete eligible analytic cohort. Eligibility criteria were applied sequentially without reference to HF/NYHA status; each excluded patient is counted once, under the first criterion not met. All 224 eligible adults admitted between 1 August 2025 and 31 July 2026 were analyzed without sampling. BMI, body mass index; HF, heart failure; NYHA, New York Heart Association; VHD, valvular heart disease. * Re-included in a sensitivity analysis (extended cohort, *n* = 260; Section 3.5).

**Table 1:** Baseline characteristics according to the registry valvular exposure.

Characteristic	VHD (*n* = 38)	No VHD (*n* = 186)	*p* Value
Age, years	63 [52.3–69.0]	62 [51.0–68.0]	0.684
Male sex	30 (78.9%)	138 (74.2%)	0.681
BMI, kg/m^2^	26.0 [23.3–27.0]	26.1 [23.5–28.2]	0.512
Documented smoking history	25 (65.8%)	118 (63.4%)	0.854
Total cholesterol, mg/dL	192.5 [172.5–236.3]	198.0 [170.0–229.0]	0.741
Explicitly documented HF (primary outcome)	24 (63.2%)	72 (38.7%)	0.007
HF/NYHA-positive (sensitivity outcome)	28 (73.7%)	101 (54.3%)	0.031
NYHA-only positivity	4 (10.5%)	29 (15.6%)	0.615

Data are median [IQR] or *n* (%) for all 224 eligible patients. Continuous variables were compared using Mann–Whitney U tests and categorical variables using Fisher’s exact tests. Explicitly documented HF is the primary outcome; the combined HF/NYHA-positive field is an over-inclusive sensitivity outcome. BMI, body mass index; HF, heart failure; NYHA, New York Heart Association; VHD, valvular heart disease.

**Table 2:** Logistic regression for explicitly documented heart failure (primary outcome).

Outcome/Variable	Unit/Reference	OR	95% CI	*p* Value
Primary explicit HF: unadjusted VHD	VHD vs. no VHD	2.71	1.32–5.59	0.007
Primary explicit HF: adjusted VHD	VHD vs. no VHD	2.87	1.38–5.99	0.005
Primary explicit HF: age	Per 10 years	0.96	0.77–1.19	0.684
Primary explicit HF: male sex	Male vs. female	1.73	0.77–3.86	0.182
Primary explicit HF: BMI	Per 5 kg/m^2^	1.02	0.72–1.44	0.912
Primary explicit HF: smoking history	Yes vs. no	1.08	0.62–1.89	0.781
Primary explicit HF: total cholesterol	Per 10 mg/dL	0.97	0.91–1.03	0.287
Combined sensitivity: unadjusted VHD	VHD vs. no VHD	2.36	1.08–5.13	0.031
Combined sensitivity: adjusted VHD	VHD vs. no VHD	2.38	1.09–5.21	0.030

The primary model included the registry VHD exposure, age, sex, BMI, documented smoking history, and total cholesterol; all covariate estimates are displayed. The combined HF/NYHA-positive sensitivity analysis used the same adjustment set and also detailed in Table S2. Major cardiovascular and renal confounders were unavailable; adjusted ORs therefore must not be interpreted as independent or causal effects. CI, confidence interval; HF, heart failure; OR, odds ratio; VHD, valvular heart disease.

## Data Availability

De-identified clinical and laboratory data supporting the findings of this study are available from the corresponding author upon reasonable request, subject to institutional data-sharing policies.

## Supplementary Materials

The following supporting information can be downloaded at: https://www.mdpi.com/article/10.3390/medicina62101835/s1, Table S1: STROBE checklist for cross-sectional studies; Table S2: Heart-failure ascertainment categories and sensitivity analyses.

## Abbreviations

The following abbreviations are used in this manuscript:

BMI	Body mass index
CI	Confidence interval
HF	Heart failure
HFmrEF	Heart failure with mildly reduced ejection fraction
HFpEF	Heart failure with preserved ejection fraction
HFrEF	Heart failure with reduced ejection fraction
IQR	Interquartile range
NYHA	New York Heart Association
OR	Odds ratio
STROBE	Strengthening the Reporting of Observational Studies in Epidemiology
VHD	Valvular heart disease
