# Supplementary material for: Documented Valvular Regurgitation and Explicit Heart Failure in an Internal Medicine Registry: A Cross-Sectional Study
Source: Medicina (Kaunas). 2026 Sep 23;62(10):1835. doi: 10.3390/medicina62101835 (PMC13707230; doi:10.3390/medicina62101835)
Supplement: Supplementary file 1 [file medicina-62-01835-s001.zip › medicina-4576404-supplementary.pdf]

Supplementary Materials

Table S1. STROBE checklist for cross-sectional studies

#	Item	Recommendation	Reported in	Status
1a	Title and abstract	Indicate the study's design with a commonly used term in the title or the abstract	Title ("A Cross-Sectional Study"); Abstract; Keywords	Yes
1b	Title and abstract	Provide in the abstract an informative and balanced summary of what was done and what was found	Abstract	Yes
2	Background/rationale	Explain the scientific background and rationale for the investigation being reported	Introduction, paragraphs 1–3	Yes
3	Objectives	State specific objectives, including any prespecified hypotheses	Introduction, paragraph 4	Yes
4	Study design	Present key elements of study design early in the paper	Section 2.1; Abstract	Yes
5	Setting	Describe the setting, locations, and relevant dates, including periods of recruitment, exposure, follow-up, and data collection	Sections 2.1 and 2.7 (Internal Medicine II, Clinical CF Hospital Timișoara; patients admitted 1 August 2025 – 31 July 2026)	Yes
6a	Participants	Give the eligibility criteria, and the sources and methods of selection of participants	Sections 2.2, 2.3; Figure 1	Yes
7	Variables	Clearly define all outcomes, exposures, predictors, potential confounders, and effect modifiers. Give diagnostic criteria, if applicable	Exposure: Section 2.3 (observed exposure exclusively documented regurgitation). Primary outcome: Section 2.4 (explicit HF). Covariates: Section 2.5. Unavailable confounders: Section 2.6	Yes
8	Data sources/ measurement	For each variable of interest, give sources of data and details of methods of assessment. Describe comparability of assessment methods if there is more than one group	Sections 2.3–2.5. Assessment identical across groups; absence of standardized echocardiographic adjudication stated in Section 2.3	Yes
9	Bias	Describe any efforts to address potential sources of bias	Sections 2.2–2.4; Sections 3.4, 3.5, 4.2 and 4.3; Table S2 (outcome-definition and cholesterol-exclusion sensitivity analyses)	Yes

#	Item	Recommendation	Reported in	Status
10	Study size	Explain how the study size was arrived at	Section 2.2 (no a priori power calculation; study size determined by the complete eligible cohort: 312 screened, 88 excluded, 224 analyzed—38 VHD and 186 non-VHD patients)	Yes
11	Quantitative variables	Explain how quantitative variables were handled in the analyses. If applicable, describe which groupings were chosen and why	Sections 2.5, 2.6. All continuous variables kept continuous and scaled per unit; no categorization	Yes
12a	Statistical methods	Describe all statistical methods, including those used to control for confounding	Section 2.6	Yes
12b	Statistical methods	Describe any methods used to examine subgroups and interactions	Not applicable — no subgroup or interaction analyses performed; rationale for not attempting an etiologic subgroup analysis in Section 4.1	N/A
12c	Statistical methods	Explain how missing data were addressed	Sections 2.2 and 2.6. Patients with missing or implausible analysis variables were excluded, with counts by criterion (Section 2.2; Figure 1); no imputation was performed. The 36 patients excluded only for total cholesterol were re-included in a sensitivity analysis (Section 3.5; Table S2, Panel C)	Yes
12d	Statistical methods	If applicable, describe analytical methods taking account of sampling strategy	Not applicable — complete eligible cohort; no sampling was performed (Section 2.2)	N/A
12e	Statistical methods	Describe any sensitivity analyses	Section 2.6 (combined HF/NYHA outcome sensitivity analysis; re-inclusion of patients excluded for total cholesterol); Sections 3.4 and 3.5; Table S2	Yes
13a	Participants	Report numbers of individuals at each stage of the study	Section 2.2; Section 3.1; Figure 1 (312 screened, 88 excluded, 224 analyzed: 38 VHD, 186 non-VHD)	Yes
13b	Participants	Give reasons for non-participation at each stage	Section 2.2; Figure 1 (missing consent, n = 14;	Yes

#	Item	Recommendation	Reported in	Status
			uninterpretable VHD or HF/NYHA fields, n = 18; implausible/missing BMI, n = 11; uninterpretable smoking code, n = 9; missing or out-of-range total cholesterol, n = 36)	
13c	Participants	Consider use of a flow diagram	Figure 1 (participant flow diagram from screening to analysis)	Yes
14a	Descriptive data	Give characteristics of study participants and information on exposures and potential confounders	Section 3.1; Table 1	Yes
14b	Descriptive data	Indicate number of participants with missing data for each variable of interest	Sections 2.2 and 2.6; Figure 1. Patients with missing analysis variables were excluded and counted by criterion; no analysis variable had missing values in the analytic cohort	Yes
15	Outcome data	Report numbers of outcome events or summary measures	Section 3.2; Table 1 (primary explicit-HF and combined sensitivity outcomes)	Yes
16a	Main results	Give unadjusted estimates and, if applicable, confounder-adjusted estimates and their precision. Make clear which confounders were adjusted for and why they were included	Section 3.3; Table 2 (primary explicit-HF model). Covariate selection justified in Section 2.6	Yes
16b	Main results	Report category boundaries when continuous variables were categorized	Not applicable — no continuous variable was categorized	N/A
16c	Main results	If relevant, consider translating estimates of relative risk into absolute risk for a meaningful time period	Section 3.2 and Table 1 report stratum-specific explicit-HF frequencies (63.2% vs. 38.7%); no time-based absolute risk is derivable from a cross-sectional design	Yes
17	Other analyses	Report other analyses done — e.g. analyses of subgroups and interactions, and sensitivity analyses	Sections 3.4–3.5; Table S2 (combined outcome and cholesterol-exclusion sensitivity analyses)	Yes
18	Key results	Summarise key results with reference to study objectives	Section 4, opening paragraph; Section 5	Yes
19	Limitations	Discuss limitations of the study, taking into account sources of potential bias or imprecision. Discuss both	Sections 4.2 and 4.3	Yes

#	Item	Recommendation	Reported in	Status
		direction and magnitude of any potential bias		
20	Interpretation	Give a cautious overall interpretation of results considering objectives, limitations, multiplicity of analyses, results from similar studies, and other relevant evidence	Sections 4, 4.1, 4.4; Section 5	Yes
21	Generalisability	Discuss the generalisability (external validity) of the study results	Section 4.3	Yes
22	Funding	Give the source of funding and the role of the funders for the present study and, if applicable, for the original study on which the present article is based	Funding statement	Yes

STROBE, Strengthening the Reporting of Observational Studies in Epidemiology. Checklist items are reproduced from von Elm et al. (reference 8 of the main text).

Table S2. Heart-failure ascertainment categories and sensitivity analyses

Panel A. Observed heart-failure ascertainment categories

Outcome category	VHD (*n* = 38)	No VHD (*n* = 186)
Explicitly documented HF	24 (63.2%)	72 (38.7%)
NYHA-only positivity	4 (10.5%)	29 (15.6%)
Original HF/NYHA-positive outcome	28 (73.7%)	101 (54.3%)
Neither explicit HF nor NYHA positivity	10 (26.3%)	85 (45.7%)

Panel B. Logistic regression for the combined HF/NYHA-positive sensitivity outcome

Combined HF/NYHA sensitivity analysis	OR	95% CI	*p* value
Unadjusted VHD	2.36	1.08–5.13	0.031
Adjusted VHD	2.38	1.09–5.21	0.030
Adjusted: age, per 10 years	0.99	0.79–1.23	0.908
Adjusted: male vs. female	0.74	0.34–1.61	0.441
Adjusted: BMI, per 5 kg/m^2	1.13	0.80–1.59	0.493
Adjusted: smoking history, yes vs. no	1.06	0.61–1.83	0.847
Adjusted: total cholesterol, per 10 mg/dL	0.99	0.93–1.06	0.857

Percentages in Panel A are column percentages. Explicit HF required a formally recorded HF diagnosis and was the primary outcome. Patients with NYHA-only positivity remained primary-outcome negative but were included as positive in the combined sensitivity outcome. In Panel B, the adjusted sensitivity model included VHD, age, sex,

BMI, documented smoking history, and total cholesterol; all covariate estimates are shown. CI, confidence interval; HF, heart failure; NYHA, New York Heart Association; OR, odds ratio; VHD, valvular heart disease.

Panel C. Sensitivity analysis including patients excluded for total cholesterol

**Extended cohort (*n* = 260)**	**VHD (*n* = 44)**	**No VHD (*n* = 216)**
Explicitly documented HF (primary outcome)	27 (61.4%)	82 (38.0%)
Combined HF/NYHA-positive sensitivity outcome	32 (72.7%)	115 (53.2%)

**Outcome / model**	**Cohort**	**OR**	**95% CI**	***p* value**
Explicit HF: unadjusted VHD	n = 260	2.60	1.33–5.05	0.005
Combined sensitivity: unadjusted VHD	n = 260	2.34	1.15–4.79	0.020

Panel C: the extended cohort comprises the 224 patients of the primary analysis plus the 36 patients excluded only for missing or out-of-range total cholesterol (Section 2.2; Figure 1). Estimates are unadjusted odds ratios from binary logistic regression and were calculated directly from the observed 2 × 2 cell counts. Confidence intervals are Wald 95% confidence intervals, and p values are two-sided Wald p values. CI, confidence interval; HF, heart failure; OR, odds ratio; VHD, valvular heart disease.
